# Calcium‐ and voltage‐driven atrial alternans: Insight from [Ca]_i_ and *V*_m_ asynchrony

**DOI:** 10.14814/phy2.15703

**Published:** 2023-05-24

**Authors:** G. Kanaporis, E. Martinez‐Hernandez, L. A. Blatter

**Affiliations:** ^1^ Department of Physiology & Biophysics Rush University Medical Center Chicago Illinois USA

**Keywords:** atrial myocyte, cardiac alternans, excitation–contraction coupling, intracellular [Ca]_i_, membrane potential

## Abstract

Cardiac alternans is defined as beat‐to‐beat alternations in contraction strength, action potential duration (APD), and Ca transient (CaT) amplitude. Cardiac excitation–contraction coupling relies on the activity of two bidirectionally coupled excitable systems, membrane voltage (*V*_m_) and Ca release. Alternans has been classified as *V*_m_‐ or Ca‐driven, depending whether a disturbance of *V*_m_ or [Ca]_i_ regulation drives the alternans. We determined the primary driver of pacing induced alternans in rabbit atrial myocytes, using combined patch clamp and fluorescence [Ca]_i_ and *V*_m_ measurements. APD and CaT alternans are typically synchronized; however, uncoupling between APD and CaT regulation can lead to CaT alternans in the absence of APD alternans, and APD alternans can fail to precipitate CaT alternans, suggesting a considerable degree of independence of CaT and APD alternans. Using alternans AP voltage clamp protocols with extra APs showed that most frequently the pre‐existing CaT alternans pattern prevailed after the extra‐beat, indicating that alternans is Ca‐driven. In electrically coupled cell pairs, dyssynchrony of APD and CaT alternans points to autonomous regulation of CaT alternans. Thus, with three novel experimental protocols, we collected evidence for Ca‐driven alternans; however, the intimately intertwined regulation of *V*_m_ and [Ca]_i_ precludes entirely independent development of CaT and APD alternans.

## 1 INTRODUCTION

Alternans is a recognized risk factor for cardiac arrhythmia—including atrial fibrillation (AF), the most common cardiac arrhythmia—and sudden cardiac death (Comtois & Nattel, 2012; Franz et al., 2012; Walker & Rosenbaum, 2003). At the cellular level, cardiac alternans is defined as beat‐to‐beat alternations in contraction amplitude (mechanical alternans), action potential duration (APD or electrical alternans), and Ca transient amplitude (CaT alternans) at constant stimulation frequency. Its cause is multifactorial (for reviews, see Blatter et al., 2003; Edwards & Blatter, 2014; Eisner et al., 2006; Kanaporis & Blatter, 2017a; Qu & Weiss, 2023; Weiss et al., 2006, 2011) which is the reason why hitherto a comprehensive and unifying paradigm of cardiac alternans has not been established. A key to the understanding of alternans mechanism is that the regulation of [Ca]_i_ and *V*_m_ is bidirectionally coupled ([Ca]_i_↔*V*_m_ coupling) and governed by complex overlapping feedback pathways. *V*_m_ → [Ca]_i_ coupling is determined by APD restitution and activity of voltage dependent ion channels that affect Ca signaling and its disturbances result in *V*_m_‐driven alternans. [Ca]_i_ → *V*_m_ coupling is determined by the effect of [Ca]_i_ dynamics, Ca fluxes, and Ca‐dependent ion currents and transporters on *V*_m_ and APD. Disturbances of [Ca]_i_ → *V*_m_ coupling lead to Ca‐driven alternans. Despite enormous progress made, it has remained a matter of debate whether a disturbance of [Ca]_i_ signaling ([Ca]_i_ → *V*_m_ coupling) or a disturbance of *V*_m_ and AP regulation (*V*_m_ → [Ca]_i_ coupling) is the primary cause of alternans (this question has indeed been referred to in the literature as the “chicken or egg” conundrum of cardiac alternans; Qu & Weiss, 2007). While there is evidence supporting both directions of coupling, recent progress (including our own: Banach & Blatter, 2023; Kanaporis & Blatter, 2015; Shkryl et al., 2012) and computational findings increasingly point to Ca signaling disturbances as the primary cause of alternans (Bien et al., 2006; Eisner et al., 2006; Goldhaber et al., 2005; Nivala & Qu, 2012; Qu et al., 2013; Rovetti et al., 2010). However, the debate is far from settled (Blatter et al., 2021; Kanaporis & Blatter, 2017a) and significant gaps in our understanding of the [Ca]_i_↔*V*_m_ interplay remain.

In general, APD and CaT alternans are coupled and coincide in time; however, we have shown previously with AP voltage clamp experiments that CaT alternans can occur in the absence of APD alternans, and pacing induced APD alternans disappeared when Ca release was inhibited (Kanaporis & Blatter, 2015), thus indicating that the temporal correlation is not absolute and the possibility of CaT and APD alternans dyssynchrony exists. Based on these observations, we set out to test whether conditions of dyssynchrony of CaT and APD alternans provide a window into the question of the primary disturbance of cellular signaling that causes atrial alternans. We conducted three sets of experiments: We investigated the onset, temporal development and termination of APD and CaT alternans in current‐clamped myocytes, we applied “extra‐beats” alternans AP voltage clamp protocols and tested their potential, efficacy or failure to disturb CaT alternans, and finally, we investigated the synchrony/dyssynchrony of CaT and APD alternans in pairs of atrial myocytes. The results indicate that the observed patterns of CaT‐APD alternans dyssynchrony support the hypothesis that atrial APD and CaT alternans can develop independently and in many instances are Ca‐driven; however, the data do not exclude the possibility that *V*_m_ disturbances have the potential to precipitate CaT alternans.

## 2 METHODS

### 2.1 Ethics statement

All procedures and protocols were approved by the Institutional Animal Care and Use Committee of Rush University Chicago.

### 2.2 Myocyte isolation

Atrial myocytes were isolated from male New Zealand White rabbits (2.5–2.9 kg; 39 rabbits; Envigo). Rabbits are the animal model of choice because cellular calcium regulation and electrophysiology are similar to that found in human myocytes, and many cardiac pathologies resemble closely human disease (Hasenfuss, 1998; Milani‐Nejad & Janssen, 2014; Panfilov, 2006; Zaragoza et al., 2011). Rabbits were anesthetized with an intravenous injection of sodium pentobarbital (Euthasol; 100 mg/kg) and heparin (1000 IU/kg). The depth of the anesthesia was evaluated by foot pinch or checking corneal reflexes. Hearts were excised, mounted on a Langendorff apparatus, and retrogradely perfused via the aorta. After an initial 5–10 min perfusion with oxygenated Ca‐free Tyrode solution (in mM: 140 NaCl, 4 KCl, 10 D‐Glucose, 5 Hepes, 1 MgCl_2_, 1000 IU/L Heparin; pH 7.4 with NaOH), the heart was perfused with minimal essential medium Eagle (MEM) solution containing 20 μM Ca and 22.5 μg/mL Liberase TH (Sigma‐Aldrich) for ~20 min at 37°C. The left atrium was dissected from the heart and minced, filtered and washed in MEM solution containing 50 μM Ca and 10 mg/mL bovine serum albumin. Isolated cells were washed and kept in MEM solution with 50 μM Ca at room temperature (20–24°C) and were used within 1–8 h after isolation.

### 2.3 Chemicals and solutions

During experiments, cells were superfused continuously with external Tyrode solution composed of (mM): 135 NaCl, 5 KCl, 2 CaCl_2_, 1 MgCl_2_, 10 Hepes, 10 D‐glucose; pH 7.4 with NaOH. All chemicals and reagents were from Sigma‐Aldrich unless otherwise stated.

### 2.4 Fluorescent [Ca]_i_ measurements

The fluorescent Ca‐sensitive probes Indo‐1 and Fluo‐4 were used to measure dynamic changes of [Ca]_i_. In current‐ and voltage‐clamp experiments Fluo‐4 or Indo‐1 pentapotassium salts were added to the pipette solution (see below). For simultaneous optical *V*_m_ and [Ca]_i_ measurements in cell pairs [Ca]_i_ was recorded with Rhod‐2 (see below). During the course of experiments, cells were continuously superfused with Tyrode solution. Fluo‐4 was excited at 485 nm with a Xe arc lamp and signals were collected at 515 nm using a photomultiplier tube. Background‐subtracted fluorescence emission signals (F) were normalized to diastolic or resting fluorescence (F_0_), and changes of [Ca]_i_ are presented as changes of F/F_0_. Indo‐1 fluorescence was excited at 357 nm (Xe arc lamp) and emitted cellular fluorescence was recorded simultaneously at 410 nm (F_410_) and 485 nm (F_485_) with photomultiplier tubes. F_410_ and F_485_ signals were background subtracted and changes of [Ca]_i_ were expressed as changes of the ratio *R* = F_410_/F_485_. Data recording and digitization were achieved using the Axon Digidata 1440A interface and pCLAMP 10.7 software (Molecular Devices). Fluorescence signals were low‐pass filtered at 30 Hz.

### 2.5 Simultaneous optical AP and [Ca]_i_ measurements

Simultaneous *V*_m_ and [Ca]_i_ measurements in atrial cell pairs were conducted with fluorescent indicators. For *V*_m_ measurements, the voltage‐sensitive fluorescent probe FluoVolt (Martinez‐Hernandez et al., 2022; McPheeters et al., 2017) was used and [Ca]_i_ was recorded with the membrane‐permeable Ca indicator Rhod‐2/AM (both Thermo Fisher Scientific). Confocal microscopy (Nikon A1R, Nikon Corporation) was used for APs and [Ca]_i_ imaging. Cells were loaded for 15 min with FluoVolt in standard Tyrode solution using proprietary (Thermo Fisher Scientific) loading conditions and dye concentration, followed by 10 min wash. Both FluoVolt™ dye (component A of the Thermo Fisher loading kit) and FluoVolt™ Loading Solution (component B) had a final concentration of 1X (specific concentrations not disclosed by manufacturer). FluoVolt preloaded atrial myocytes were subsequently loaded for 15 min with 5 μM Rhod‐2/AM in the presence of 0.25% Pluronic F‐127. Cells were washed with Tyrode solution and preincubated with blebbistatin (10 μM) for 5 min to minimize motion artifacts. FluoVolt was excited at 488 nm and emission collected at wavelengths >515 nm while Rhod‐2 am was excited at 543 nm and emission collected at wavelengths >600 nm. FluoVolt and Rhod‐2 signals are presented as *F*/*F*_0_ ratios where *F*_0_ represents resting *V*_m_ and [Ca]_i_ at the beginning of a recording. [Ca]_i_ and optical AP measurements were conducted in the confocal line scan mode (512 lines/s) using a ×40 oil‐immersion objective lens. The scan line was placed along the transverse axis of the cell avoiding the nucleus. CaTs and APs were elicited by electrical field stimulation of intact atrial myocytes using a pair of platinum electrodes (5 ms voltage pulses set at ∼50% above the threshold for triggering APs and CaTs). In this set of experiments, cell pairs were stimulated at 1.4 Hz. Electrical coupling between cells was confirmed by monitoring simultaneous spontaneous depolarizations of both cells during a 10‐s period of rest immediately following cessation of field stimulation. While the exact degree of electrical coupling and the intercellular resistance are unknown in these experiments, the simultaneous occurrence of *V*_m_ depolarization in both cells of the magnitude of an AP and the similarity of the time course of the spontaneous depolarizations were taken as a strong indication of robust electrical coupling. Experiments were conducted at room temperature (22–24°C).

### 2.6 Patch clamp experiments

Patch clamp pipettes (1.5–3 MΩ filled with internal solution) were pulled from borosilicate glass capillaries (WPI) with a horizontal puller (model P‐97; Sutter Instruments). Pipettes were filled with an internal solution containing (mM): 130 K glutamate, 10 NaCl, 10 KCl, 0.33 MgCl_2_, 4 MgATP, and 10 Hepes with pH adjusted to 7.2 with KOH. For simultaneous *V*_m_ and [Ca]_i_ measurements, 100 μM Fluo‐4 pentapotassium salt or 75 μM Indo‐1 pentapotassium salt (Thermo Fisher Scientific) was added to the internal solution. The internal solution was filtered through 0.22 μm pore filters. Electrophysiological signals were recorded from single atrial myocytes in the whole‐cell ruptured patch clamp configuration using an Axopatch 200A patch clamp amplifier, the Axon Digidata 1440A interface, and pCLAMP 10.7 software (Molecular Devices). AP recordings were low‐pass filtered at 5 kHz and digitized at 10 kHz. All patch clamp experiments were performed at room temperature (20–24°C).

For AP measurements, the whole‐cell “fast” current clamp mode of the Axopatch 200A was used and APs were evoked by 4 ms stimulation pulses with a magnitude ~1.5 times higher than AP activation threshold. *V*_m_ measurements were corrected for a junction potential error of −10 mV.

**FIGURE 1:**
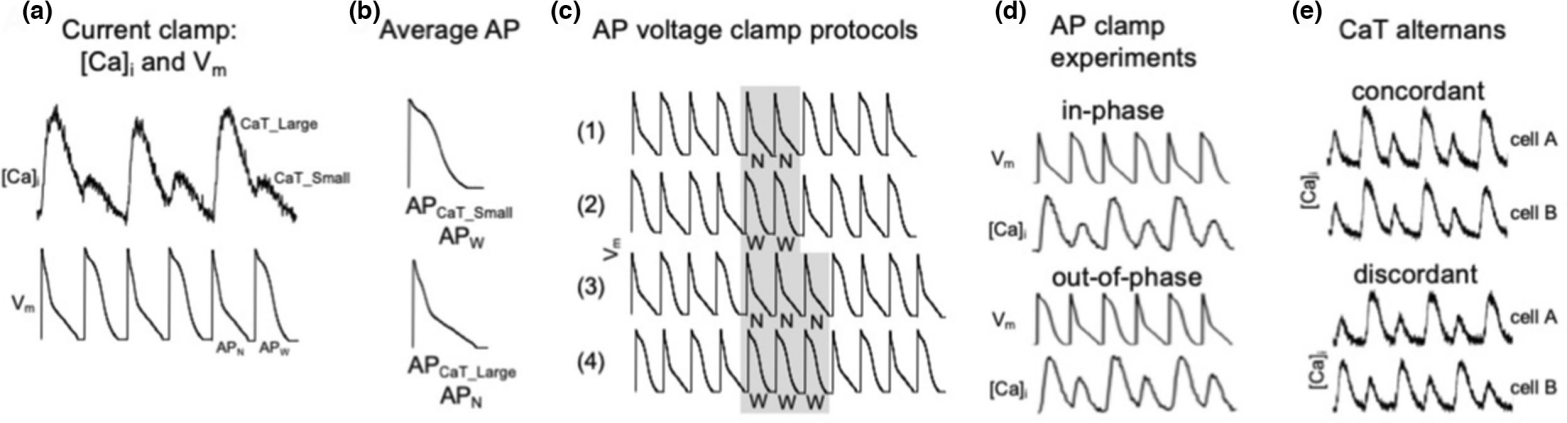
Terminology and experimental protocols. (a) Simultaneous CaT and APD alternans measurement in current clamped rabbit atrial myocyte. APD_N_: APD recorded during the large amplitude alternans CaT; APD_W_: APD recorded during the small amplitude alternans CaT. (b) Average APD_CaT_Small_/APD_W_ and average APD_CaT_Large_/APD_N_ constructed from experiments as illustrated in (a). To construct the average AP, three consecutive APs recorded from three individual cells paced at 1.3 Hz and exhibiting CaT alternans were averaged. (c) Command voltages for four different AP alternans voltage clamp protocols to deliver extra beats. N: APD_CaT_Large_ or APD_N_; W: APD_CaT_Small_ or APD_W_. (d) AP alternans voltage clamp experiments result in either ‘in‐phase’ or ‘out‐of‐phase’ CaT alternans. “In‐phase” refers to the situation typically occurring in current clamped cells (i.e., when the voltage is not clamped and is controlled by the cell; cf. panel a). (e) CaT alternans in cell pairs. Concordant: small and large amplitude CaTs coincide in both cells; Discordant: small amplitude CaT in cell A coincides with large amplitude CaT in cell B and vice versa.

For AP voltage clamp experiments, voltage commands in form of atrial APs were generated from averages of APs (three consecutive APs/cell) recorded in current‐clamp experiments from three individual cells paced at 1.3 Hz and exhibiting CaT alternans (Kanaporis & Blatter, 2015, 2017b; Figure 1a,b). These representative AP waveforms were used in all AP voltage clamp experiments. Thus, the AP voltage commands were not specific for an individual cell and therefore are expected to differ marginally from the endogenous AP of a specific cell. Two voltage commands were generated: AP_CaT_Large_ representing APs recorded during large amplitude alternans CaTs, here also referred to as AP_N_ (where “N” stands for “Narrow”). AP_CaT_Small_ refers to the AP‐waveform observed during small amplitude alternans CaTs, also termed here AP_W_ (“W” for “Wide”). These two distinct AP waveforms were used to generate the following four pacing protocols (Figure 1c) (i) alternans AP clamp interrupted by two consecutive AP_N_ (…N‐W‐N‐W‐**N‐N**‐W‐N‐W‐N …; referred to here as “NN‐protocol”); (ii) alternans AP clamp interrupted by two consecutive AP_W_ (…W‐N‐W‐N‐**W‐W**‐N‐W‐N‐W…; WW‐protocol); (iii) alternans AP clamp interrupted by three consecutive AP_N_ (…N‐W‐N‐W‐**N‐N‐N**‐W‐N‐W‐N…; NNN‐protocol); and (iv) alternans AP clamp interrupted by three consecutive AP_W_ (…W‐N‐W‐N‐**W‐W‐W**‐N‐W‐N‐W…; WWW‐protocol). Stimulation frequency was modified by varying the cycle length (CL) between 840 and 390 ms by changing diastolic intervals between voltage commands (CLs applied: 390, 440, 490, 540, 640, and 840 ms). In alternans AP voltage clamp experiments, when AP_CaT_Small_/AP_W_ elicited a small amplitude CaT and AP_CaT_Large_/AP_N_ elicited a large amplitude CaT (as expected from current clamp experiments where AP kinetics were determined entirely by the cell), CaT and APD alternans are termed “in‐phase” (Kanaporis & Blatter, 2017b). This was the case for 244 of a total of 303 experimental observations (81%) in this study. Less frequently (19%), AP_W_ elicited a large amplitude CaT and AP_N_ elicited a small amplitude CaT. In this case, CaT and APD alternans are termed “out‐of‐phase” (Figure 1d).

### 2.7 CaT alternans

APD and CaT alternans were induced by electrical pacing, either by electrical field stimulation or by a depolarizing pulse in current clamp and AP voltage clamp experiments. Typical range where stable CaT alternans was observed was 1.2–2.5 Hz. In current clamp experiments, CaT alternans was induced by incrementally increasing the pacing frequency from a basal pacing frequency of 0.5 or 1 Hz until stable alternans was observed (stimulation frequency increments: 1.3, 1.6, 1.8, 2 and 2.5 Hz). The degree of CaT alternans was quantified as the alternans ratio (AR). AR = 1−[Ca]_i,Small_/[Ca]_i,Large_, where [Ca]_i,Large_ and [Ca]_i, Small_ are the amplitudes of the large and small CaTs of a pair of alternating CaTs. By this definition, AR values fall between 0 and 1, where AR = 0 indicates no CaT alternans and AR = 1 indicates a situation where sarcoplasmic reticulum (SR) Ca release is completely abolished on every other beat. CaTs were considered alternating when the beat‐to‐beat difference in CaT amplitude exceeded 10% or AR >0.1 (Kanaporis & Blatter, 2015).

For CaT alternans measurements in cell pairs, alternans are termed concordant when large and small amplitude CaTs, respectively, coincide in both cells (Figure 1e). Alternans is termed discordant when the small amplitude CaT in one cell coincides with the large amplitude CaT in the other cell, and vice versa. Analogously, APD alternans is concordant when AP_W_ and AP_N_ coincide in both cells, while during discordant APD alternans AP_W_ in one cell coincides with AP_N_ in the other cell, and during the subsequent beat AP_N_ in the first cell coincides with AP_W_ in the second cell.

## 3 RESULTS

### 3.1 Kinetics and synchrony/dyssynchrony of onset and termination of CaT and APD alternans

**FIGURE 2:**
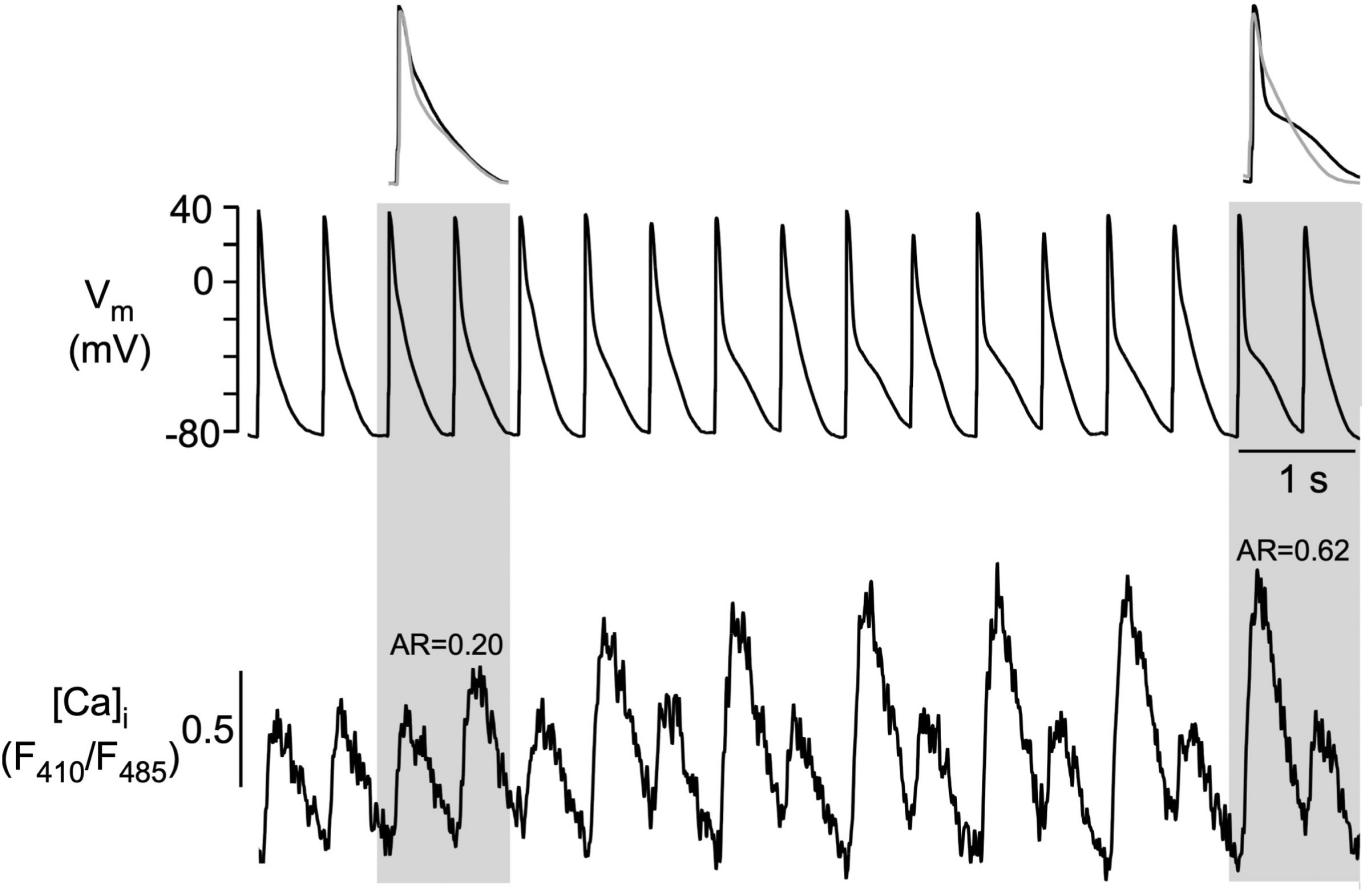
Synchronized APD and CaT alternans in single rabbit atrial myocyte. Simultaneous recording of APs (current clamp) and CaTs, showing initiation of pacing induced alternans. *n* = 40 observations of a total of 52 observations. Top: overlays of two consecutive APs recorded at time intervals marked by the gray bars. [Ca]_i_ was measured with Indo‐1. AR, alternans ratio.

**FIGURE 3:**
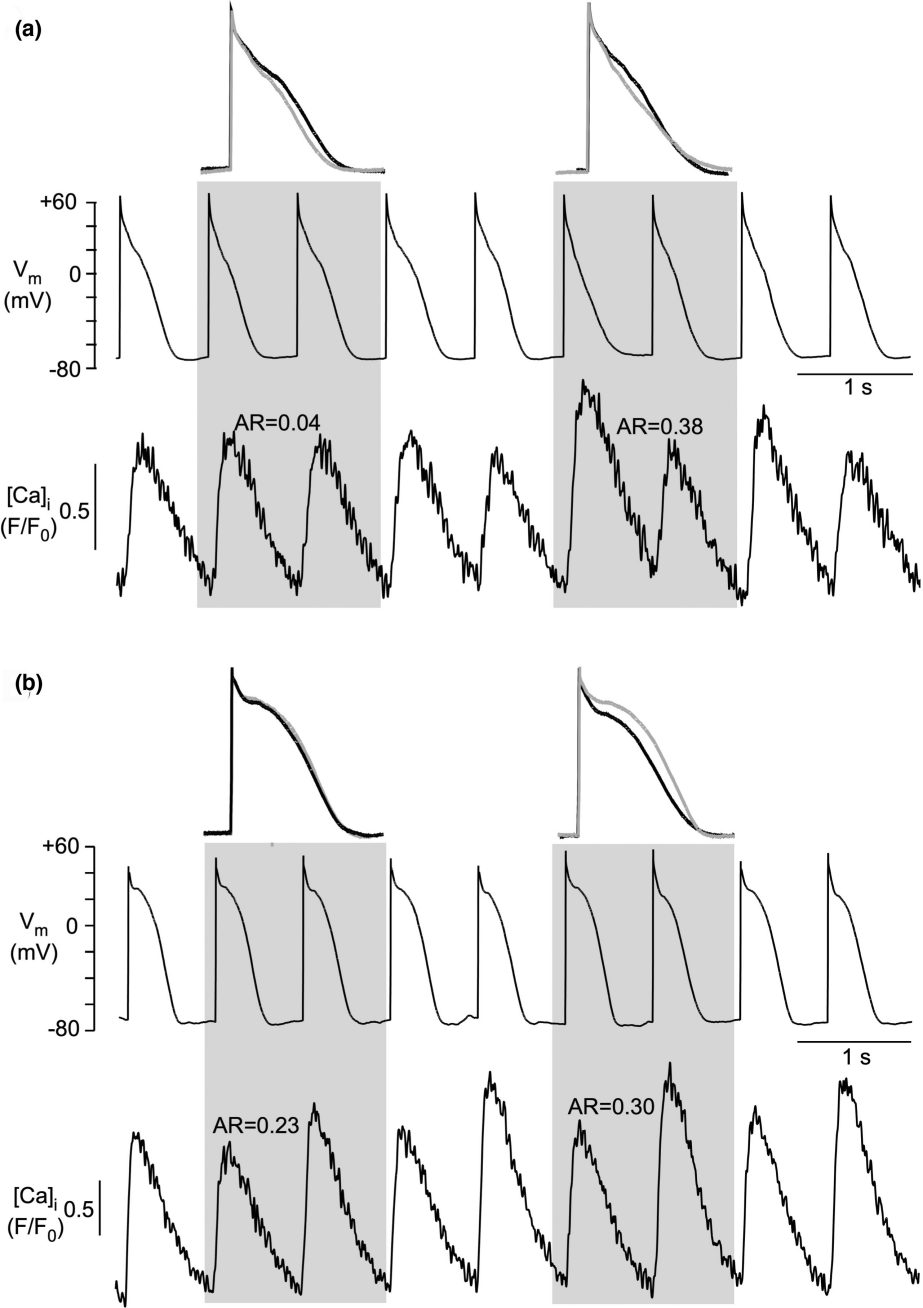
Simultaneous APD (current clamp) and CaT alternans recording in single rabbit atrial myocyte. (a) APD alternans onset precedes CaT alternans. *n* = 5 observations of a total of 52 observations. (b) CaT alternans onset precedes APD alternans. *n* = 7 observations of a total of 52 observations. Top in both panels: overlay of two consecutive APs recorded at time intervals marked by the gray bars. [Ca]_i_ was measured with Fluo‐4. AR, alternans ratio.

In a first set of experiments, we investigated the onset and termination kinetics of pacing‐induced CaT and APD alternans (*N* = 16 rabbits; *n* = 24 cells). Experiments were conducted in Fluo‐4 or Indo‐1 loaded cells under current clamp conditions to measure [Ca]_i_ and *V*_m_ simultaneously. The aim of this part of the study was to gain insight from the detailed investigation of onset and termination kinetics of atrial alternans whether alternans were Ca‐ or *V*_m_‐driven. Specifically, we were interested whether CaT and APD alternans started and terminated synchronously or whether there was a temporal uncoupling of CaT and APD alternans start and termination. Figure 2 shows an example of highly synchronized onset of CaT and APD alternans, that is, the onset occurred on the same beat, illustrated by the overlays of two consecutive APs recorded at the times indicated by the gray bar. In 76.9% of the observations, APD and CaT alternans started simultaneously; thus, a synchronized onset was the most commonly observed scenario (alternans was defined to be present when AR >0.1). However, while common, the high degree of synchronization of alternans onset was not always present and dyssynchrony between CaT and APD alternans lasting for one or more beats was observed. Figure 3a shows an example where begin of APD alternans preceded CaT alternans (9.6% of observations), whereas Figure 3b shows the opposite where CaT alternans started before APD alternans (13.5% of observations). These observations suggest that both APD alternans and CaT alternans can develop independently.

**FIGURE 4:**
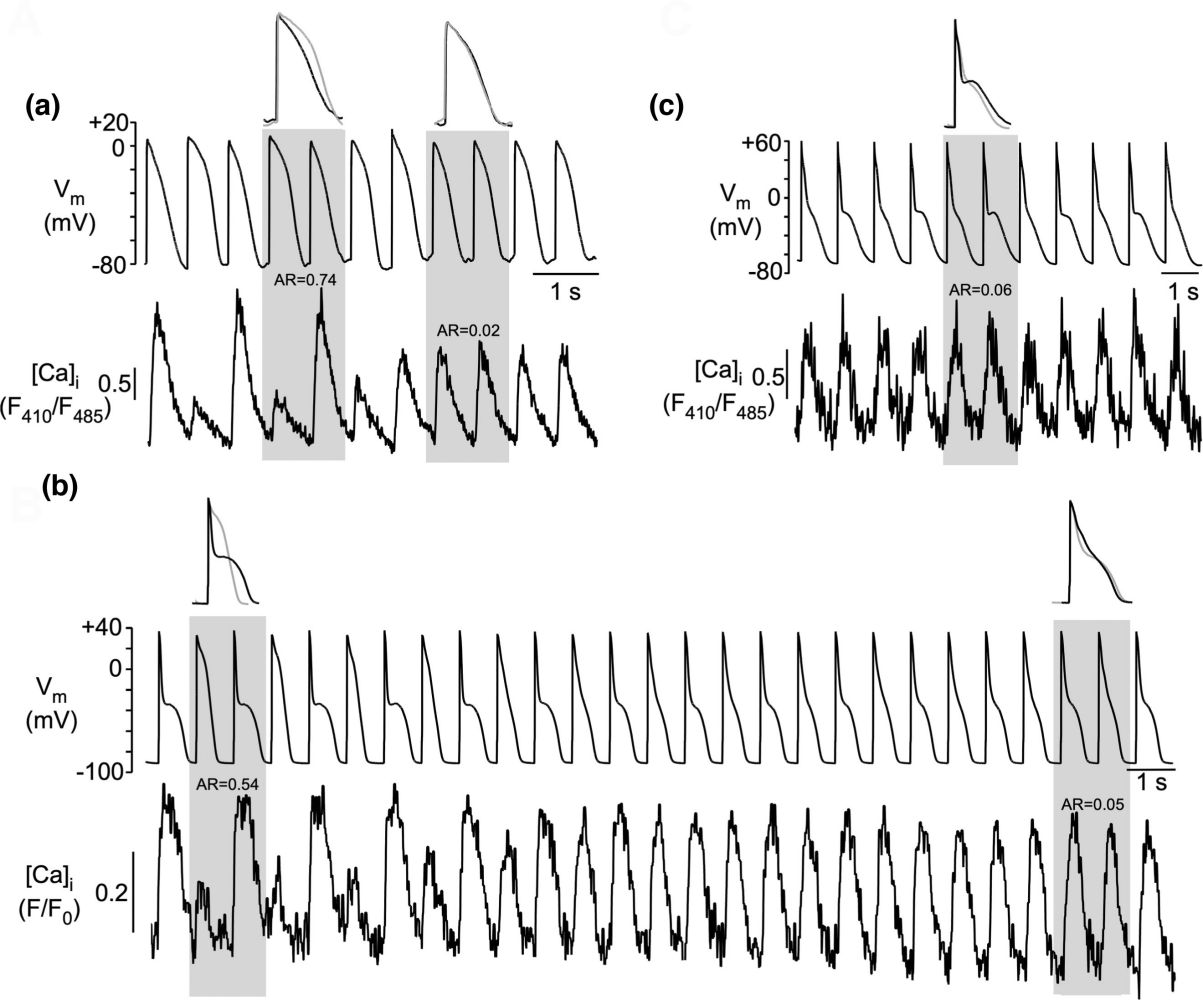
Simultaneous APD (current clamp) and CaT alternans recording in single rabbit atrial myocyte. (a) Simultaneous termination of APD and CaT alternans. *n* = 4 observations of a total of 11 observations. (b) Dyssynchronous termination of APD and CaT alternans. APD alternans continues after cessation of CaT alternans. *n* = 7 observations of a total of 11 observations. (c) Prolonged episode of APD alternans in the absence of CaT alternans. Top in all panels: overlay of two consecutive APs recorded at time intervals marked by the gray bars. [Ca]_i_ was measured with Indo‐1 (panels a and c) or Fluo‐4 (panel b). AR, alternans ratio.

We gained further valuable insight into the synchrony/dyssynchrony dynamics of CaT and APD alternans through closer examination of alternans termination kinetics. As previously mentioned, overall APD and CaT alternans are synchronized, including termination of alternans, as illustrated in Figure 4a. However, synchrony between CaT and APD alternans termination was not an obligatory feature and variable degrees of dyssynchrony between APD and CaT alternans termination were observed. Figure 4b shows an example where APD alternans continued for >15 beats after cessation of CaT alternans, and Figure 4c shows an example where pronounced APD alternans failed to be accompanied by CaT alternans. Furthermore, we did not observe the situation where APD alternans terminated before cessation of CaT alternans, that is, CaT alternans terminated either simultaneously with APD alternans (36% of observations) or in the majority of the cases before (64%), but never after APD alternans termination. These observations support the hypothesis that alternans onset and termination are primarily (but not exclusively, Figure 3a) Ca‐driven.

### 3.2 Effect of extra‐beats on synchrony/dyssynchrony of CaT and APD alternans

In the next set of experiments, we tested the effect of extra‐beat stimulations on alternans AP‐clamped atrial myocytes (*N* = 12 rabbits). Atrial myocytes were voltage clamped with an AP alternans command voltage, using actual AP morphologies previously recorded during pacing induced CaT alternans in atrial myocytes (for details, see Methods section; Figure 1). Four different AP voltage clamp protocols were applied. With two protocols, the overall phase of APD alternans remained unchanged and only the shape of a single AP was changed from wide (W) to narrow (N) or from N to W (NNN‐ and WWW‐protocol; Figure 1c). The other two protocols introduced a phase shift in the sequence of APD alternans command voltage by adding a single extra‐beat (NN‐ and WW‐protocol).

**FIGURE 5:**
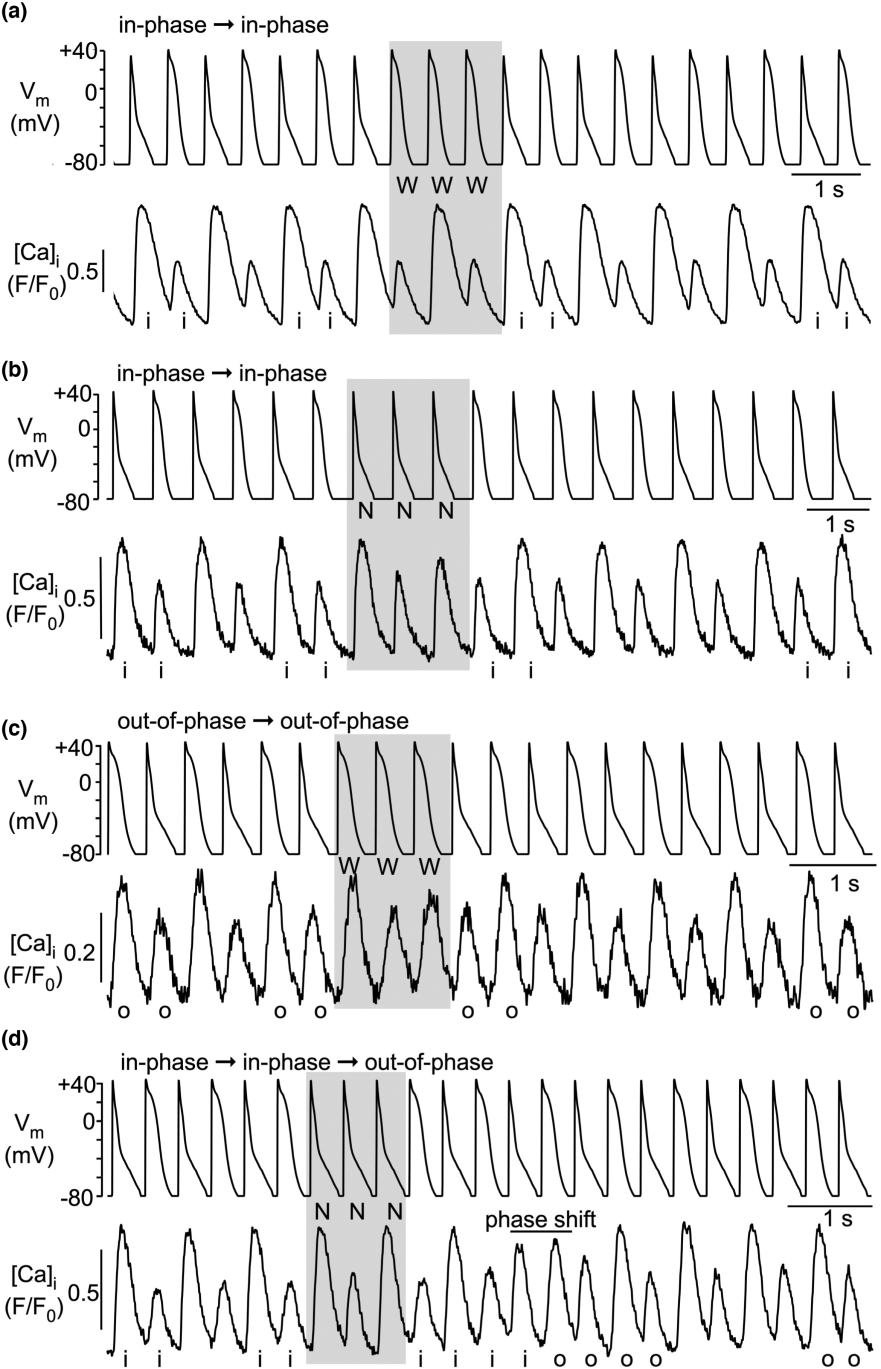
CaT alternans in AP alternans voltage‐clamped atrial myocytes elicited with WWW‐ and NNN‐protocols. (a) WWW‐protocol applied to in‐phase CaT alternans failed to induce a phase shift. (b) NNN‐protocol applied to in‐phase CaT alternans failed to induce a phase shift. (c) WWW‐protocol applied to out‐of‐phase CaT alternans failed to induce a phase shift. (d) NNN‐protocol applied to in‐phase CaT alternans caused phase shift from in‐phase to out‐of‐phase alternans. i: in‐phase; o: out‐of‐phase. [Ca]_i_ was measured with Fluo‐4. Number of recordings with WWW‐protocol: 70. Number of recordings with NNN‐protocol: 81.

A total of 151 recordings with the WWW‐ and the NNN‐protocols were analyzed (WWW‐protocol: *n* = 13 cells; NNN‐protocol; *n* = 15 cells). The vast majority of recordings showed that CaT and APD alternans were in‐phase before the intervention and remained in‐phase (134 of 151 recordings; 89%; Figure 5a,b). In 9% of recordings (13/151), alternans was out‐of‐phase before the intervention and remained out‐of‐phase (Figure 5c). On rather rare occasions (4/151; <3%), the intervention caused a phase shift, either from in‐phase to out‐of‐phase or from out‐of‐phase to in‐phase. An example is shown in Figure 5d. Overall, these experiments (Figure 5) show that changing the morphology of a single AP, while maintaining the overall phase of APD alternans, has very limited or no effect on the course of concomitant CaT alternans, suggesting that CaT alternans are regulated rather autonomously.

**FIGURE 6:**
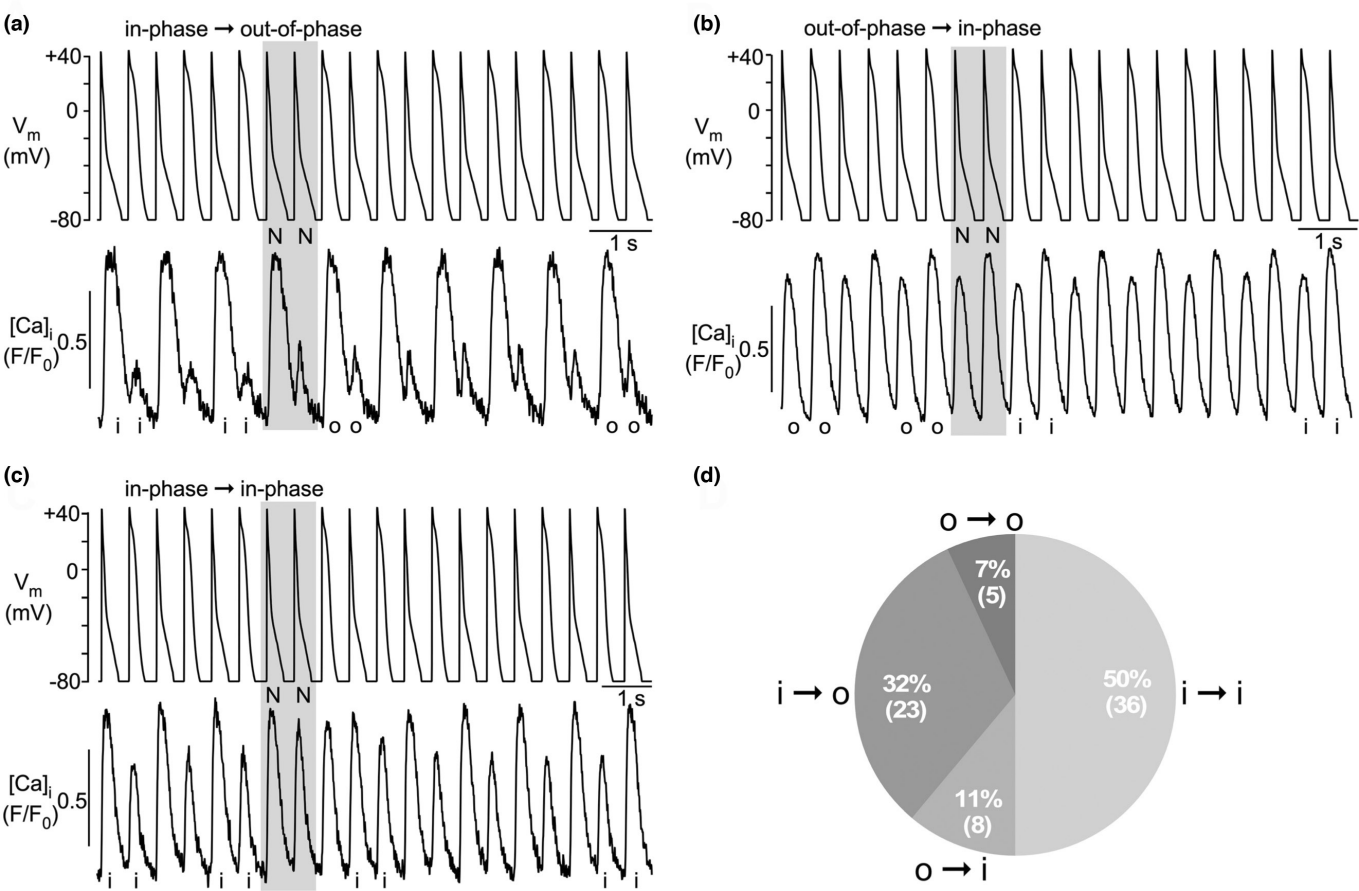
CaT alternans in AP alternans voltage‐clamped atrial myocytes elicited with NN‐protocol. (a) NN‐protocol applied to in‐phase CaT alternans caused phase shift from in‐phase to out‐of‐phase alternans. (b) NN‐protocol applied to out‐of‐phase CaT alternans caused phase shift from out‐of‐phase to in‐phase alternans. (c) NN‐protocol applied to in‐phase CaT alternans failed to induce a phase shift. (d) Frequency distribution of effect of NN‐protocol on alternans phase. i: in‐phase; o: out‐of‐phase. [Ca]_i_ was measured with Fluo‐4. Number of recordings with NN‐protocol: 72.

**FIGURE 7:**
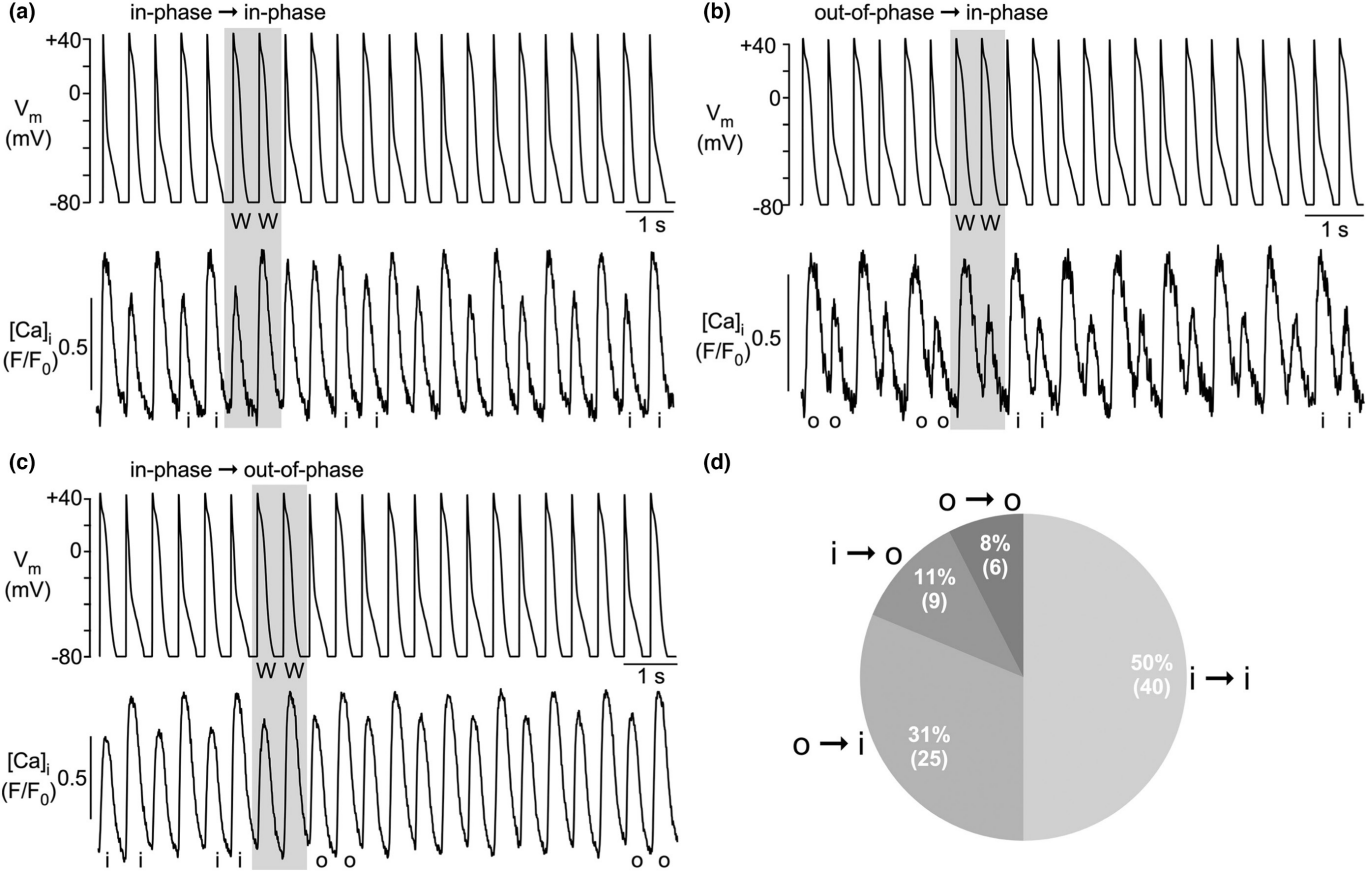
CaT alternans in AP alternans voltage‐clamped atrial myocytes elicited with WW‐protocol. (a) WW‐protocol applied to in‐phase CaT alternans failed to induce a phase shift. (b) WW‐protocol applied to out‐of‐phase CaT alternans caused phase shift from out‐of‐phase to in‐phase alternans. (c) WW‐protocol applied to in‐phase CaT alternans caused phase shift from in‐phase to out‐of‐phase alternans. (d) Frequency distribution of effect of WW‐protocol on alternans phase. i, in‐phase; o, out‐of‐phase. [Ca]_i_ was measured with Fluo‐4. Number of recordings with WW‐protocol: 80.

Clearly more profound effects on the course of CaT alternans could be achieved by the protocols that introduced a phase shift of APD alternans (NN‐protocol, Figure 6; WW‐protocol, Figure 7). Of a total of 72 experiments where the NN‐protocol was applied (*n* = 19 cells), in 59 (82%) experiments CaT alternans started in‐phase (i), and 13 (18%) experiments started out‐of‐phase (o). During application of the NN‐protocol, in 57% of recordings (41/72), the APD phase shift failed to shift the phase of the CaT alternans (Figure 6c), whereas in 43% of the experiments (31/72), the APD phase shift was accompanied by a phase shift in CaT alternans (i → o or o → i). When a CaT alternans phase shift occurred, it was more commonly from in‐phase to out‐of‐phase (32% of all NN‐experiments; Figure 6a) and less common from out‐of‐phase to in‐phase (11% of all NN‐experiments; Figure 6b). Of all experiments that started in‐phase (59 experiments) 61% remained in‐phase (36/59) and only 39% (23/59) shifted to out‐of‐phase, supporting the notion that in‐phase is the preferred condition. Similarly, of all experiments that started out‐of‐phase (13 experiments) only 38% (5/13) stayed out‐of‐phase, while the majority (62%; 8/13) changed to the preferred in‐phase condition. The effect of the APD phase shift induced by the NN‐protocol is summarized in Figure 6d.

**FIGURE 8:**
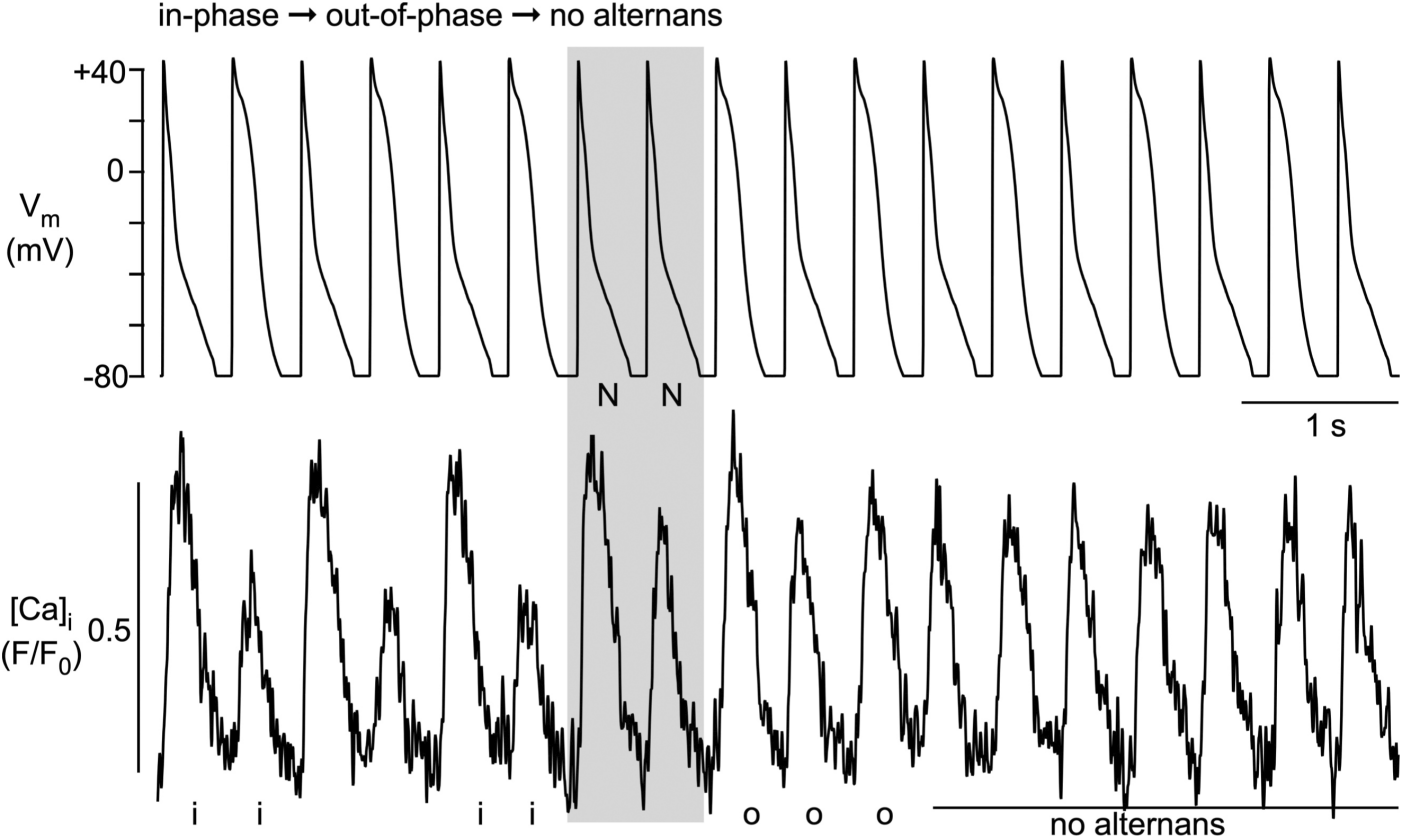
CaT alternans in AP alternans voltage‐clamped atrial myocytes elicited with NN‐protocol. NN‐protocol applied to in‐phase CaT alternans caused a transient phase shift from in‐phase to out‐of‐phase, followed by cessation of CaT alternans. i: in‐phase; o: out‐of‐phase. [Ca]_i_ was measured with Fluo‐4. *n* = 6 observations.

Similar effects were achieved with the WW‐protocol (*n* = 20 cells). In 58% of recordings (46/80), the APD phase shift failed to change the phase of CaT alternans (Figure 7a,d). While in these cases, a phase shift was absent, occasionally a transient period of CaT amplitude irregularities that could last for several beats (Figure 7a) could be observed. These CaT disturbances typically consisted of irregular CaT amplitudes with no clear alternans pattern. A CaT alternans phase shift was observed in 42% of recordings (34/80) and was more common for out‐of‐phase to in‐phase (25/80, 31%; Figure 7b) than for in‐phase to out‐of‐phase (9/80, 11%; Figure 7c). Again, the data confirm that “in‐phase” is the preferred condition. Of all experiments that started out‐of‐phase (31 experiments) only 19% (6/31) stayed out‐of‐phase, while 81% (25/31) changed to in‐phase. Of all experiments that started in‐phase (49 experiments) 82% (40/49) stayed in‐phase and only 18% (9/49) changed to out‐of‐phase. Finally, in a small fraction of experiments (6 observations), application of the NN‐ or WW‐protocol caused a cessation of CaT alternans after the intervention (Figure 8). In the example shown, application of the NN‐protocol caused a phase shift from in‐phase to out‐of‐phase followed by cessation of CaT alternans.

In summary, these experiments showed that simply changing the shape of a single AP during APD alternans (NNN‐ and WWW‐protocol) had little or no effect on CaT alternans; thus in these cases, the overall alternans pattern remained unchanged. More profound were the effects of a phase shift in APD alternans (NN‐ and WW‐protocol) on the course of CaT alternans. The following trends were observed: (i) in most cases (108/152), CaT and APD alternans were in‐phase before the intervention and remained in‐phase after the intervention (76/108), that is, the in‐phase pattern, which is physiologically observed, overall remains the preferred pattern also in these specific voltage clamp experiments. Considering all experiments, essentially an identical fraction of experiments (109/152) showed in‐phase CaT alternans after the intervention, irrespective whether a phase shift had occurred or not. (ii) A phase shift of CaT alternans (i → o and o → i) was observed in 65 of a total of 152 recordings. For a phase shift of CaT to occur with the NN‐ and WW‐protocols, requires that the large‐small‐large‐small amplitude pattern of CaT alternans is maintained and thus argues for a largely autonomous regulation of CaT alternans and a high degree of independence from APD alternans. (iii) Interestingly, in all recordings with a CaT alternans phase shift 43% of the cells revealed an AR >0.5. In stark contrast, in experiments without a CaT alternans phase shift less than 8% of the cells had an AR >0.5. Thus, prominent CaT alternans is a factor that facilitates a phase shift of CaT alternans (e.g., Figure 6a) and resulted in an undisturbed continuation of the CaT alternans pattern after the application of the extra beat.

### 3.3 Synchrony/dyssynchrony of CaT and APD alternans in cell pairs

**FIGURE 9:**
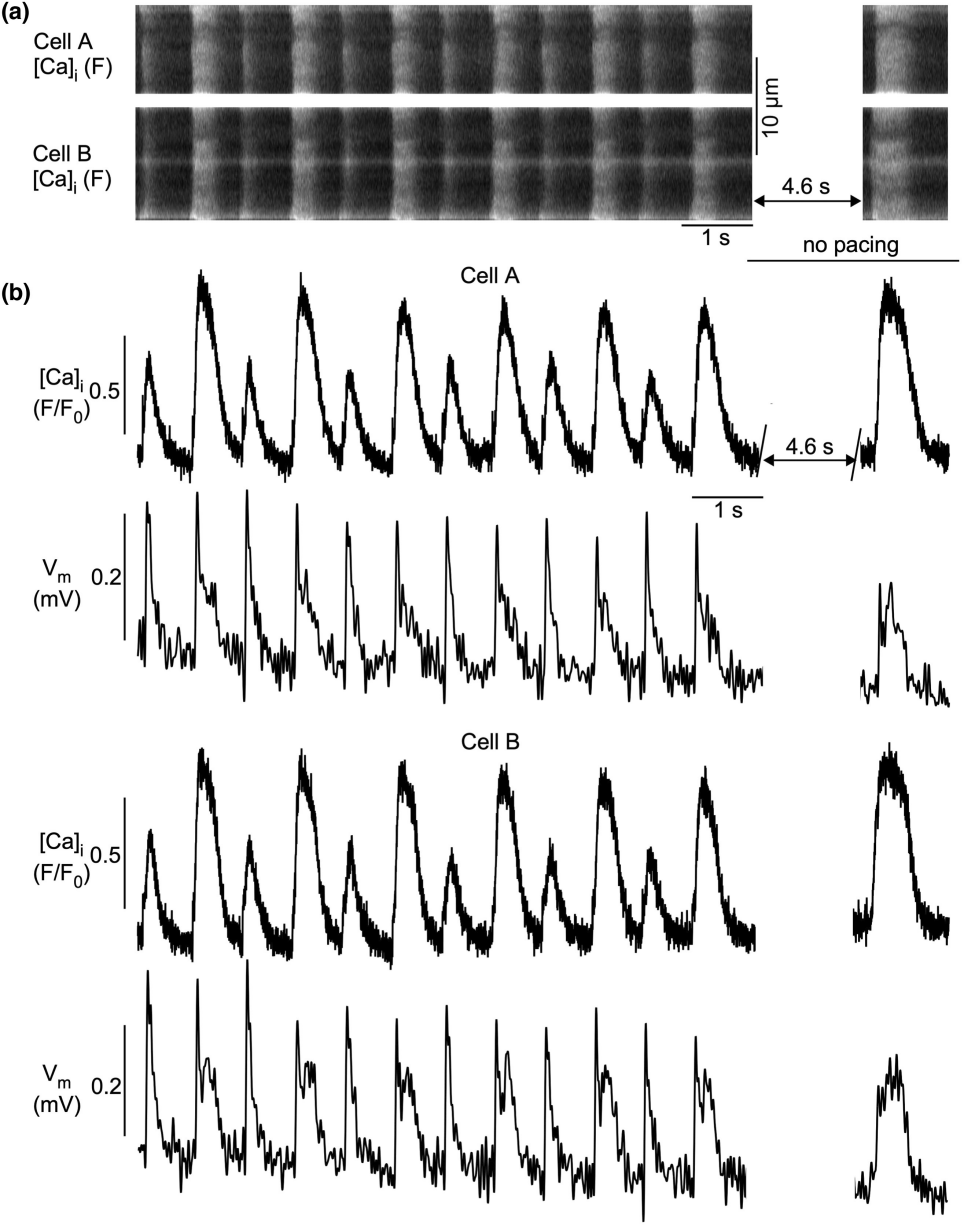
Simultaneous measurements of CaT and APD alternans in atrial myocyte pairs. *V*_m_ and [Ca]_i_ were recorded with fluorescence transverse line scan confocal imaging. *V*_m_ probe: FluoVolt; Ca probe: Rhod‐2. (a) Confocal line scan (x‐t) [Ca]_i_ (Rhod‐2 fluorescence) images. (b) [Ca]_i_ and *V*_m_ traces recorded from line scan images in panel (a). Signals were spatially averaged over the width of the cell. Highly synchronized concordant CaT and APD alternans were recorded. Synchronized spontaneous *V*_m_ depolarization and Ca release during rest after pacing indicate electrical coupling between the two cells. Number of cell pairs with synchronized APD and CaT alternans: 40.

We next investigated the spatio‐temporal organization of APD and CaT alternans in atrial cell pairs (*N* = 11 rabbits; *n* = 37 cell pairs). [Ca]_i_ and *V*_m_ were measured simultaneously using the fluorescent probes Rhod‐2 and FluoVolt, respectively. Fluorescence signals were recorded in confocal transverse line scan mode (Figure 9a). Alternans was induced by electrical pacing by field stimulation (1.4 Hz). Most commonly, both CaT and APD alternans were concordant in electrically coupled cell pairs, as illustrated in Figure 9. Figure 9a shows line scan (x‐t) images of the two cells. Figure 9b shows [Ca]_i_ and *V*_m_ signals derived from the line scan images and averaged over the width of the cell. To test for electrical coupling between the two cells, we took advantage of the fact that rabbit atrial myocytes showed spontaneous activity (spontaneous Ca release and spontaneous membrane depolarizations and APs) during a period of rest immediately following electrical pacing at rates that elicited alternans. As shown in Figure 9, approximately 5 s after cessation of pacing both cells showed a simultaneous spontaneous *V*_m_ depolarization that was accompanied by a transient elevation of [Ca]_i_. The simultaneous occurrence of a depolarization signal of similar magnitude and time course in both cells was taken as clear evidence of electrical coupling between the two cells of a pair.

**FIGURE 10:**
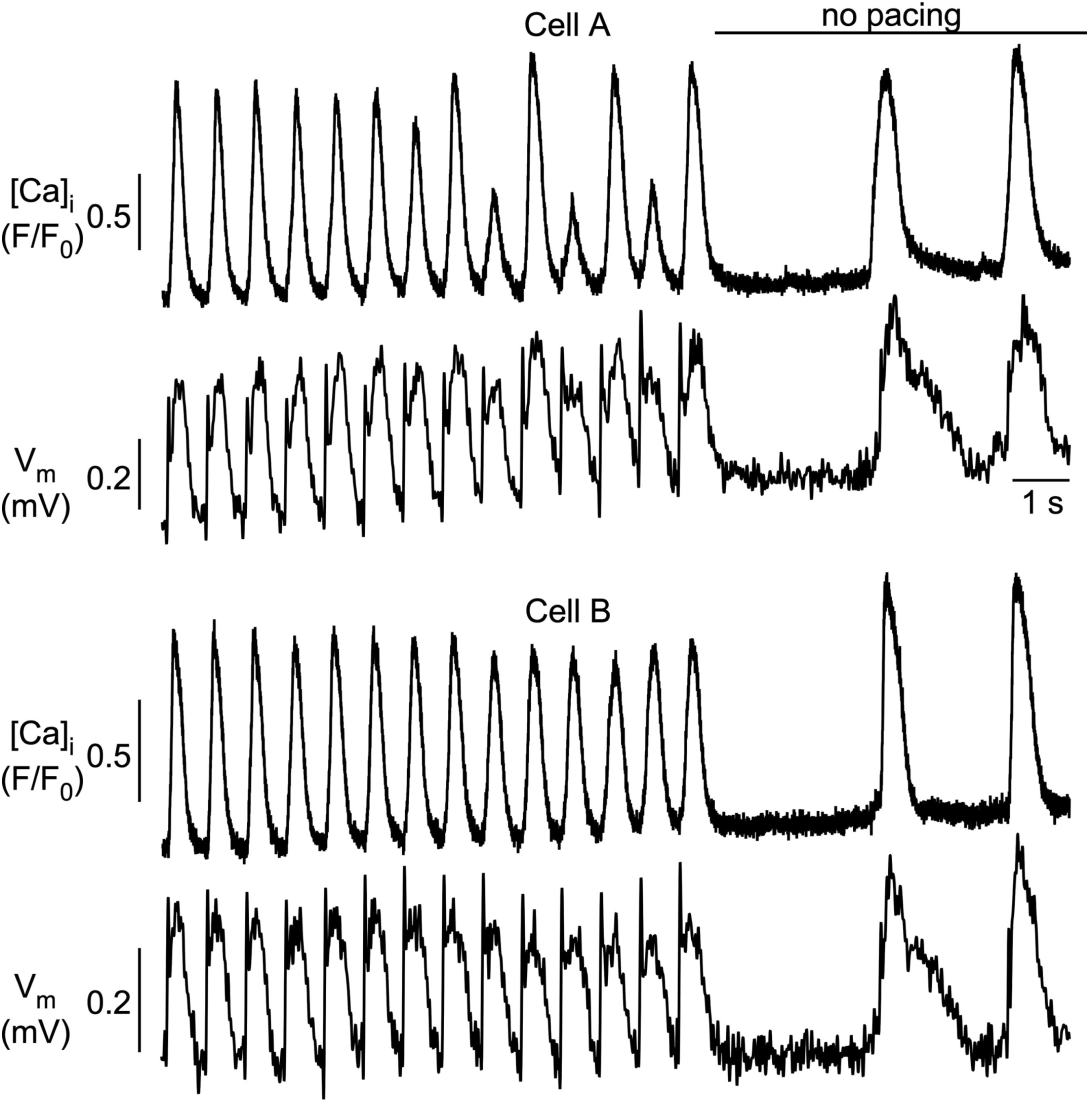
Simultaneous measurements of CaT and APD alternans in atrial myocyte pairs. *V*_m_ (FluoVolt) and [Ca]_i_ (Rhod‐2) were recorded with fluorescence transverse line scan confocal imaging. Cell A reveals the development of synchronized CaT and APD alternans. Cell B failed to develop alternans. Two synchronized spontaneous *V*_m_ depolarizations and Ca release events during rest after pacing indicate electrical coupling between the two cells. Number of cell pairs showing dyssynchrony of APD and CaT alternans: 15.

**FIGURE 11:**
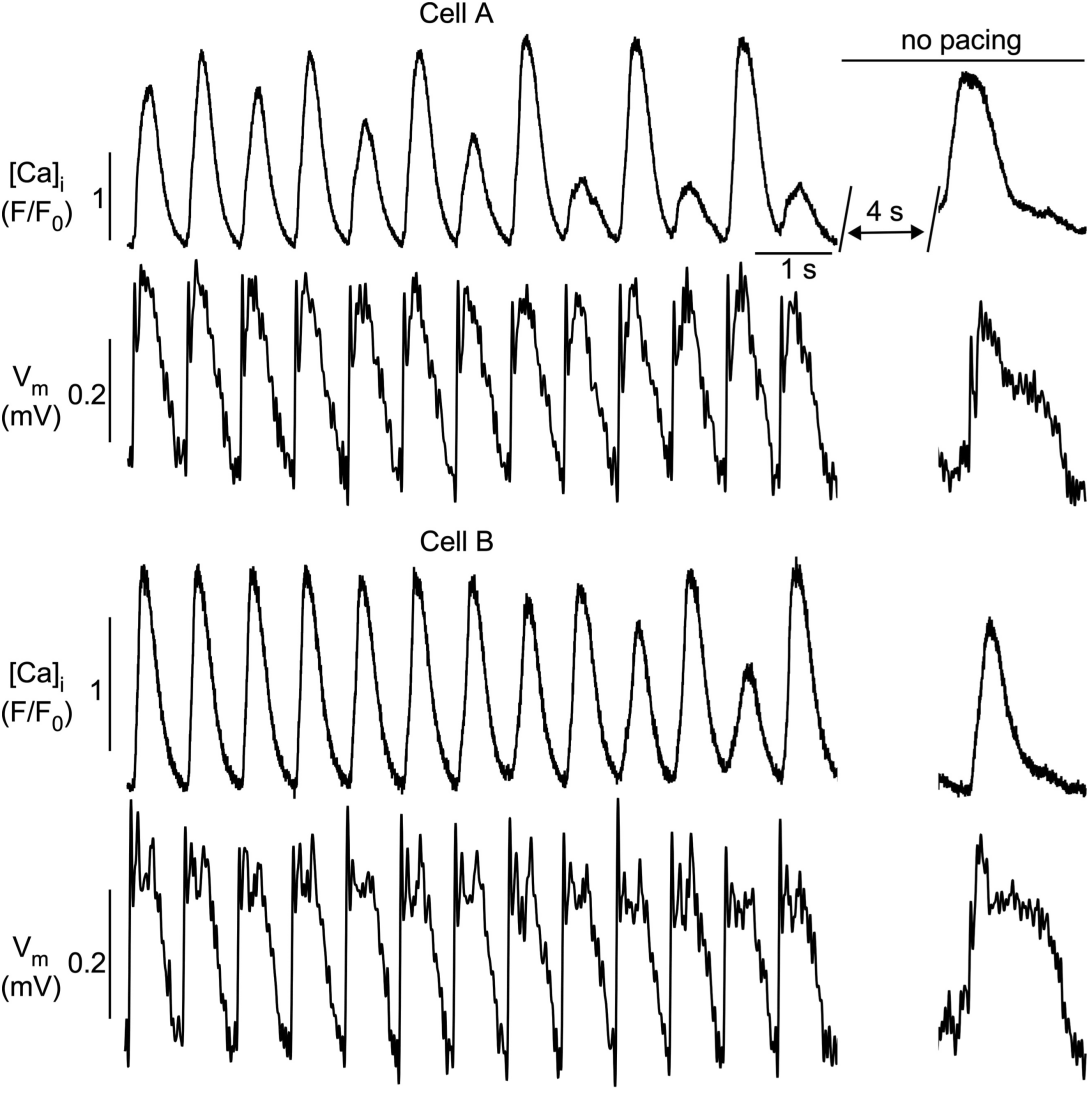
Simultaneous measurements of CaT and APD alternans in atrial myocyte pairs. *V*_m_ (FluoVolt) and [Ca]_i_ (Rhod‐2) were recorded with fluorescence transverse line scan confocal imaging. CaT alternans develop autonomously in the absence of APD alternans at different times in the two cells and are discordant. Synchronized spontaneous *V*_m_ depolarization and Ca release during rest after pacing indicate electrical coupling of the two cells. Number of cell pairs showing dyssynchrony of APD and CaT alternans: 15.

**FIGURE 12:**
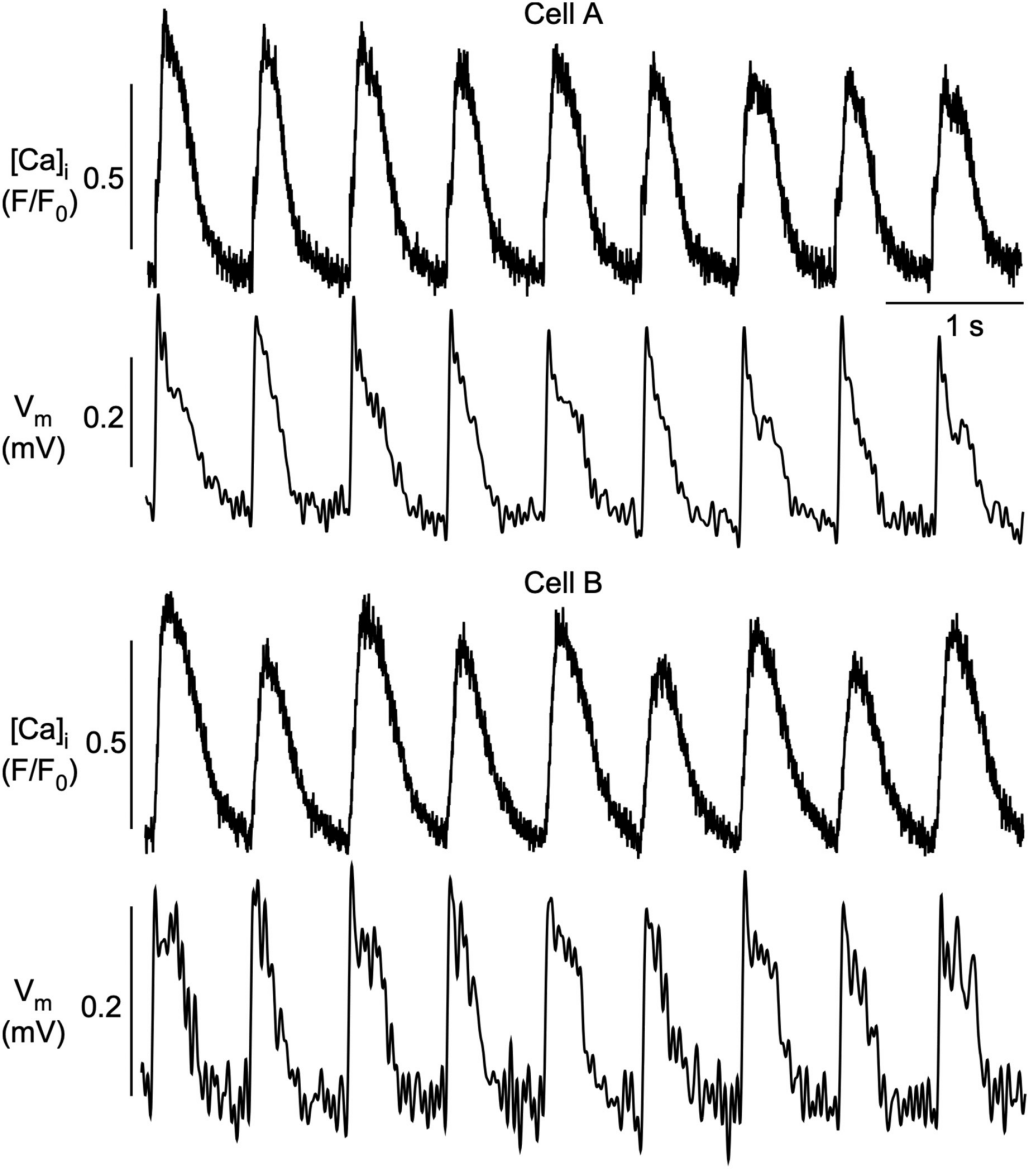
Simultaneous measurements of CaT and APD alternans in atrial myocyte pairs. *V*_m_ (FluoVolt) and [Ca]_i_ (Rhod‐2) were recorded with fluorescence transverse line scan confocal imaging. The two cells show concordant APD alternans; however, only cell B developed CaT alternans. Number of cell pairs showing dyssynchrony of APD and CaT alternans: 15.

The high degree of synchronization between [Ca]_i_ and *V*_m_ signals in form of concordant alternans was observed in the majority of experiments. However, several deviations from this general pattern was observed that provided new insight into the underlying [Ca]_i_ and *V*_m_ disturbances causing alternans. Figures 10, 11, 12 illustrate examples of dyssynchrony of APD and CaT alternans in atrial cell pairs. Figure 10 shows an example where one cell developed CaT alternans whereas in the other cell the CaT amplitudes remained constant, despite electrical coupling between the two cells. The CaT alternans in cell A was accompanied by APD alternans, that did not extend to cell B. Thus, the example illustrates dyssynchrony of CaT and APD alternans between the two cells despite electrical coupling.

Figure 11 illustrates that CaT alternans can develop autonomously and essentially in the absence of APD alternans. Both cells of the pair developed CaT alternans however with different onset. The interval between onset in the two cells lasted >7 beats. In addition, CaT alternans was discordant. Furthermore, CaT alternans developed in the apparent absence of APD alternans, indicative of dissociation of [Ca]_i_ regulation from *V*_m_ control, despite clearly evident electrical coupling between the cells.

Finally, Figure 12 shows an example of uncoupling of [Ca]_i_ and *V*_m_ regulation with different effects on alternans development, compared to Figure 11. Here, both cells show concordant APD alternans; however, APD alternans was accompanied by CaT alternans only in cell B, but not cell A.

In summary, the examples shown in Figures 10, 11, 12 indicate that CaT alternans can develop with variable degrees of independence from APD alternans and can escape complete synchronization between the two cells, despite electrical coupling between the cells. These observations again suggest a degree of autonomy of CaT alternans development and support the [Ca]_i_ → *V*_m_ coupling hypothesis for alternans.

## 4 DISCUSSION

In this study, we investigated the organization of dyssynchrony of APD and CaT alternans in order to gain insight whether atrial cellular alternans are primarily *V*_m_‐ or Ca‐driven. The main findings are: (i) in general, APD and CaT alternans are synchronized in time and magnitude, however uncoupling between APD and CaT alternans onset, duration and termination was observed; (ii) simultaneous AP and CaT measurements in current‐clamped myocytes revealed that CaT alternans can develop in the absence of APD alternans, and that APD alternans can fail to precipitate CaT alternans, suggesting independence of CaT alternans development from *V*_m_ disturbances; (iii) using an alternans AP voltage clamp protocol that included application of one extra AP (NN‐ and WW‐protocol) showed that a phase shift in the sequence of APs was accompanied by a phase shift in nearly half of the experiments, indicating that the CaT alternans pattern prevailed undisturbed and thus alternans appear to be Ca‐driven. Furthermore, in 50% of the experiments, CaT started and remained in‐phase with APD alternans after the extra AP, suggesting that the physiological APD‐CaT alternans relationship remains the preferred pattern also in AP voltage clamp experiments; (iv) while APD and CaT alternans in electrically coupled cell pairs are typically synchronized between cells, uncoupling of CaT alternans from APD alternans as well as dyssynchrony of CaT alternans between the two cells can occur, suggesting autonomous regulation of CaT alternans.

In cardiac cells, the regulation of *V*_m_ and [Ca]_i_ is intimately linked and bi‐directionally coupled. Complex feedback pathways between *V*_m_ and [Ca]_i_ regulation are governed by transmembrane ion gradients and the activity of surface membrane ion channels and transporters. Cardiac alternans is considered a proarrhythmic condition and in the atria has been causally linked to AF. CaT and APD alternans are highly correlated in time and space, as well as in the degree of alternans quantified as APD and CaT alternans ratios (Kanaporis & Blatter, 2015). Disturbances of both *V*_m_ and [Ca]_i_ regulation have been identified that facilitate the occurrence of alternans. This has led to the distinction between Ca‐ and voltage‐driven alternans (Qu & Weiss, 2023). Two main elements of cellular Ca signaling have emerged as potential causes of Ca‐driven alternans: SR Ca load and refractoriness of the SR Ca release channel, the ryanodine receptor (RyR). Ca alternans driven by SR Ca load alternans, by definition, entail a beat‐to‐beat alternating end‐diastolic filling of the SR (Diaz et al., 2004; Kanaporis & Blatter, 2017b) and facilitates CaT alternans further through the steepness of the non‐linear fractional SR Ca release function (Eisner et al., 2000; Xie et al., 2008) and limitations to the rate of diastolic refilling of the SR by SERCA. SR Ca load‐driven alternans has been observed experimentally and formulated mathematically in computational studies. For this mechanism to be able to sustain alternans beat‐to‐beat alternans of SR Ca load is an obligatory feature. However, the observation that CaT alternans can occur in the absence of SR Ca load alternans (Huser et al., 2000; Picht et al., 2006; Shkryl et al., 2012) argues in favor of additional alternans mechanisms. Refractoriness of the RyR and the SR Ca release machinery has emerged as an alternative key factor for Ca‐driven alternans (Alvarez‐Lacalle et al., 2013; Kornyeyev et al., 2012; Lugo et al., 2014; Shkryl et al., 2012). RyR refractoriness refers to the fact that SR Ca release is unavailable after a release for a limited time interval, and therefore becomes increasingly more important at shorter diastolic intervals. In contrast, at low heart rates, when the diastolic interval exceeds RyR refractoriness, the SR Ca load mechanism becomes the predominant mechanism of Ca‐driven alternans. Recently, an elegant conceptual model for cardiac alternans has been forwarded, termed “3R theory” of alternans (Cui et al., 2009; Nivala & Qu, 2012; Qu et al., 2013; Rovetti et al., 2010) that links Ca spark properties (i.e., the properties of elementary Ca release events from individual Ca release unites of the SR) to whole‐cell CaT alternans and forms the basis of a unifying theory of CaT alternans. The 3R theory predicts by numerical computations how instabilities in the relationship of three critical spark attributes (Randomness, Recruitment, Refractoriness) lead to alternans, and how excitation–contraction coupling Ca handling proteins (L‐type Ca channels, RyR, SERCA, Na/Ca exchanger, Ca buffers, and IP_3_ receptor Ca release channel) and Ca handling organelles (SR, mitochondria) determine alternans probability.

Alternatively, disturbances of *V*_m_ regulation have also been identified as cause of cardiac alternans (*V*_m_ → [Ca]_i_ coupling). Three mechanisms of *V*_m_‐driven alternans have been proposed (Qu et al., 2010). First, a steep APD restitution curve facilitates alternans (Martinez‐Hernandez et al., 2022; Nolasco & Dahlen, 1968) which becomes particularly prominent at high heart rates or short diastolic intervals. Additional conditions that facilitate *V*_m_‐driven alternans involve AP prolongation (Kanaporis et al., 2019; Kanaporis & Blatter, 2023) and, related to it, early afterdepolarizations (EADs; Qu & Weiss, 2023). However, also very short APs, brought upon by K channel activity, have been linked to *V*_m_‐driven alternans (Fish & Antzelevitch, 2008).

It is generally agreed that disturbances of both *V*_m_ and [Ca]_i_ regulation can precipitate cardiac alternans. Voltage and Ca signaling can be considered as two excitable subsystems that are bidirectionally coupled ([Ca]_i_↔*V*_m_ coupling) and their respective regulatory mechanisms are intimately intertwined. Mediators of the interactions between the two systems are the Ca‐dependence of membrane ion channels and transporters, and the voltage dependence of Ca signaling mechanisms (Weiss et al., 2006). For example, the Ca‐dependent inactivation of voltage‐gated L‐type Ca channels (LCCs) has been implicated in the determination of APD during alternans where a large CaT tends to shorten ADP through LCC inactivation. The electrogenic Na/Ca exchanger has been shown to modulate APD where during a large amplitude alternans CaT NCX activity tends to prolong the AP. To mention a few additional examples, we have shown previously that Ca‐dependent chloride channels and LCCs (Kanaporis & Blatter, 2017b) are involved in alternans (Kanaporis & Blatter, 2016a, 2016b). Also, modulation of AP morphology by several K channels has been shown to determine the development of alternans (Kanaporis et al., 2019; Kanaporis & Blatter, 2023). Thus, undoubtedly disturbance of one of the systems inadvertently leads to failures in the other. While this notion is generally accepted, the question has been raised whether there is a preference for one system to be primarily affected and to become the driver of alternans (Qu & Weiss, 2007). Our observation that CaT alternans can occur in the absence of APD alternans could be interpreted that the primary cause roots in a Ca signaling failure. In order to address this question further, we set out in this study to investigate dyssynchronies between CaT and APD alternans using novel experimental protocols.

Simultaneous recording of CaT and *V*_m_ in single current‐clamped atrial myocytes showed that most commonly CaT and APD alternans coincide, are synchronized and in‐phase (Figure 2). However, close examination of onset and termination of alternans revealed subtle signs of dyssynchrony between CaT and APD alternans. Patterns of dyssynchrony consisted of APD alternans onset before CaT alternans, CaT alternans onset before APD alternans begin, and CaT alternans termination while APD alternans continued. These patterns of CaT and APD alternans uncoupling indicate a degree of independence of CaT and APD alternans development. Furthermore, while continued APD alternans in the absence of CaT alternans was observed, we did not record prolonged CaT alternans in the absence of APD alternans in the current study. The latter suggests that APD alternans is required for CaT alternans to occur; however, the presence of APD alternans does not guarantee the occurrence of CaT alternans (Figure 4b). (Note: the apparent absence of cell averaged CaT alternans could be mimicked by subcellular out‐of‐phase CaT alternans, as shown previously (Edwards & Blatter, 2014; Kockskamper & Blatter, 2002). However, this alternans pattern is rare and analysis of spatially resolved confocal CaT alternans data (*n* = 32 cells) failed to reveal subcellular out‐of‐phase alternans in the current study). Furthermore, as we have shown previously in AP voltage clamp experiments CaT alternans can develop in the absence of APD alternans. This is consistent with our earlier observation (Kanaporis & Blatter, 2015) in AP voltage‐clamped experiments that an APD alternans voltage clamp protocol not automatically precipitates CaT alternans. Interestingly, while in current clamp experiments we did not observe long‐lasting CaT alternans in the absence of APD alternans, previously we were able to induce CaT alternans with an AP voltage clamp protocol without APD alternans. This was also observed in rabbit ventricular myocytes (Chudin et al., 1999). The latter suggests that under conditions where the cell determines AP morphology (current clamp experiments) long‐lasting CaT alternans requires concomitant APD alternans, whereas in AP voltage clamp experiments, when an AP shape is “forced” onto the cell, CaT alternans can occur in the absence of APD alternans. Taken together, these data are consistent with the hypothesis that CaT alternans develop with a noticeable degree of independence from *V*_m_.

We collected additional experimental evidence that CaT alternans developed independently and autonomously. In the extra‐beat experiments (Figures 5, 6, 7, 8), the voltage clamp protocols where only a single AP was replaced by one of opposite duration (NNN‐ and WWW‐protocol) the course of CaT alternans was typically not altered. Only in ~4% of experiments a phase‐shift was observed, that is, only in a small fraction of experiments a single beat AP disturbance was able to permanently alter the course of CaT alternans (Figure 5). In contrast, alternans AP voltage clamp protocols that entailed an APD phase shift (NN‐ and WW‐protocol), the consequences for CaT alternans were more diverse and complex. Two CaT alternans patterns stood out as most common: (i) in half of the experiments, CaT and APD alternans began in‐phase and remained in‐phase after the extra beat intervention. This indicates that the “physiological” pattern is the preferred alternans pattern that is difficult to be altered by an AP disturbance. (ii) The second most frequently case observed (Figure 6, NN‐protocol: 43%; Figure 7, WW‐protocol: 42%) was the AP phase‐shift being accompanied by a CaT phase‐shift, that is, the cell maintained its CaT alternans pattern and the small‐large‐small‐large CaT pattern continued undisturbed, arguing in favor of Ca‐driven alternans.

Further evidence for Ca‐driven alternans came from simultaneous AP and CaT measurements in electrically coupled cell pairs. While undoubtedly CaT and APD alternans were highly synchronized in cells pairs (Figure 9), occurrence of uncoupling of CaT and APD provided interesting insight into the origin of alternans. We observed several patterns of uncoupling and dyssynchrony between the two cells: (i) CaT and APD alternans in one cell were not accompanied by CaT and APD alternans in the second cells, despite clear evidence of electrical coupling between the two cells. In this case APD alternans was not capable of driving alternans in the other cell (Figure 10). (ii) Despite clear electrical coupling CaT alternans could be discordant and occur in the absence of APD alternans (Figure 11). (iii) Synchronized concordant APD alternans (Figure 12) precipitate CaT only in one cell. Taken together, the data collected from cell pairs emphasize autonomy of CaT alternans in situations where CaT and APD among a cell pair became dyssynchronized and APD alternans was either unable to drive CaT alternans or CaT alternans developed independently of APD. In addition to single‐cell studies, electrical (and CaT) alternans have been investigated extensively at tissue, organ, and organism level. The latter include numerous clinical studies on alternans in humans, and alternans was proposed to identify patients with AF substrate (Lalani et al., 2013; Narayan et al., 2011; Verrier et al., 2016) and serve as a tool for arrhythmia risk stratification. However, cardiac cell pairs have rarely been subject of alternans investigations or have focused on cell–cell propagation of Ca waves (Li et al., 2012). This strikes as quite surprising given the fact that the cardiac cell pair represents the elementary structural and functional unit of cell–cell communication in the heart. As the elementary unit of cardiac cell–cell interaction insight from alternans study in cell pairs have the potential to serve as crucial linchpin between cellular and multicellular findings in the understanding of alternans mechanisms. This study provides a first account of APD and CaT alternans mechanism in cell pairs.

In conclusion, we have initially discussed the fact that historically the alternans field has been occupied with the lingering question whether the primary defect that leads to alternans results from disturbances of *V*_m_ regulation or from Ca signaling failures. The ever‐growing body of new data on alternans at cellular and organ level have still not generated an unequivocal answer to this question, and might indicate that the view of alternans being caused by a primary disturbance of one or the other system has become obsolete and requires a new perspective. As outlined above, cardiac excitation–contraction coupling is governed by two excitable systems (Qu & Weiss, 2023), membrane voltage, and intracellular Ca signaling. The key element here is that the two systems are bidirectionally coupled and their regulation is characterized by numerous overlapping and mutually influencing feedback pathways. When the control of both systems is entirely determined by the cell and not manipulated by experimental interventions such as interference by voltage‐ and current‐clamp approaches or exogenous Ca buffers (inadvertently induced by the use of fluorescent Ca indicator dyes), the two systems never act independently and are always under mutual influence. In other words, alternans cannot be solely Ca‐ or solely *V*_m_‐driven, rather alternans are either *predominantly V*_m_‐driven or *predominantly* Ca‐driven. In either case, however, disturbances of one of the two systems have immediate consequences for the proper functioning of the other which often results in further enhancement and stabilization of alternans.

## AUTHOR CONTRIBUTIONS

G.K, E.M.‐H was involved in the collection of data. E.M.‐H., G.K., L.A.B was involved in the conception and design of the work, data analysis and interpretation, and drafting the article. All authors have approved the final version of the manuscript.

## FUNDING INFORMATION

This work was supported by the National Institutes of Health grants HL057832, HL062231, HL080101, HL101235, HL132871, HL134781, HL155762, and HL164453, and the Fondation Leducq.

## CONFLICT OF INTEREST STATEMENT

No conflicts of interest to declare.
